# Lighthouse-shaped rapid identification device for acidic electrolyzed oxidizing water: A low-cost detection method based on conductivity differences

**DOI:** 10.1371/journal.pone.0357672

**Published:** 2026-09-03

**Authors:** Yuan Mao, Wei Zheng, Qian Chen

**Affiliations:** 1 Central Sterile Supply Department, 363 Hospital, Chengdu, China; 2 Central Sterile Supply Department, Sichuan Clinical Research Center for Cancer, Sichuan Cancer Hospital &Institute, Sichuan Cancer Center, Affiliated Cancer Hospital of University of Electronic Science and Technology of China, Chengdu, China; National Chung Cheng University College of Engineering, TAIWAN

## Abstract

**Objective:**

To address the safety risk of mistaking tap water or purified water for acidic electrolyzed oxidizing water (AEOW) during manual cleaning in central sterile supply departments (CSSDs), this study aimed to develop a low-cost, rapid identification device based on electrical conductivity differences to ensure the accuracy and safety of the disinfection process.

**Methods:**

A lighthouse-shaped rapid identification device (LRID) was fabricated based on the significantly higher electrical conductivity of AEOW compared with tap water and purified water. Centered on an Arduino Nano microcontroller, the device integrates a conductivity sensor, a wireless charging module, and an LED indication system. It achieves automatic discrimination by detecting liquid electrical conductivity: the green LED remains illuminated for AEOW, while the red LED lights up for tap water or purified water. Identification accuracy, battery life, and waterproof performance were evaluated, and the satisfaction of CSSD technicians with the conventional pH test strip method and the LRID was compared.

**Results:**

The LRID achieved 100% identification accuracy (90/90) for AEOW, tap water, and purified water collected from the CSSDs of 10 hospitals. The average battery life was 83.75 ± 1.27 h, supporting a weekly charging schedule. All devices functioned normally after continuous immersion in AEOW for 30 days, confirming reliable waterproof performance. Scores from 28 CSSD technicians showed that the LRID was significantly superior to the pH test strip method in identification accuracy, ease of operation, and promotability (P < 0.001).

**Conclusion:**

This study successfully developed and validated a low-cost (approximately 20 USD), highly reliable, and easy-to-operate AEOW rapid identification device. The LRID effectively prevents disinfection failures caused by liquid misuse in CSSDs and holds significant potential for its application and promotion.

## Introduction

Acidic electrolyzed water (AEW) is an acidic aqueous solution generated by the electrolysis of sodium chloride or hydrochloric acid solutions, with hypochlorous acid (HOCl) as the primary disinfecting component [1,2]. Based on pH levels, AEW is categorized into strongly acidic electrolyzed water (pH < 2.7, available chlorine content (ACC) 20–60 mg/L) and slightly acidic electrolyzed water (pH 5.0–6.5, ACC 10–80 mg/L) [1,3–5]. Notably, in China, strongly acidic electrolyzed water is referred to as acidic electrolyzed oxidizing water (AEOW), which has slightly different key specifications (pH 2–3, oxidation-reduction potential (ORP) ≥ 1100 mV, ACC 50–70 mg/L) [2,6].

Characterized by low pH, high ORP, and the presence of chlorine-containing compounds (primarily HOCl), AEW achieves high-level disinfection efficacy through the synergistic effect of these three factors [5,7,8]. HOCl can penetrate microbial cell membranes, oxidize key metabolic systems by generating hydroxyl radicals, damage cell membranes, induce amino acid decarboxylation, react with nucleic acids, and inactivate key enzymes. High ORP alters cellular electron flow, impairs cell membranes, oxidizes sulfhydryl compounds on the cell surface, and disrupts cellular metabolic processes. Low pH not only inhibits bacterial growth but also sensitizes the bacterial outer membrane, rendering it more susceptible to HOCl penetration [5,7,9–11]. AEW exhibits excellent broad-spectrum antimicrobial activity; with a contact time of only 0.5 minutes, it achieves a log reduction value greater than 5 against various pathogenic bacteria, including *Aeromonas hydrophila, Alcaligenes faecalis, Campylobacter jejuni, Citrobacter freundii, Enterobacter aerogenes, Enterococcus faecalis, Escherichia coli, Listeria monocytogenes, Proteus vulgaris, Pseudomonas aeruginosa, Staphylococcus aureus, Vibrio parahaemolyticus,* and *Vibrio vulnificus* [7,12–18]. AEW also demonstrates high safety: no toxic reactions were observed in high-dose acute oral administration animal experiments, mutagenicity tests were negative, and local exposure caused no skin, ocular irritation, or mucosal damage. As an environmentally friendly disinfectant, its available chlorine degrades rapidly, it has extremely low toxicity to aquatic organisms, and it ultimately decomposes into water and harmless salts without causing secondary pollution [9,19–21].

The use of electrolyzed water for hand hygiene and drinking water treatment was first reported in the 1960s [22–24]. Starting from the 1980s, research and application of AEW developed rapidly in Japan. The Ministry of Health, Labour and Welfare approved strongly acidic electrolyzed water for hand and endoscope disinfection in the medical field in 1996 and 1997, respectively, and authorized both strongly acidic and slightly acidic electrolyzed water as food additives in 2002; in the same year, the U.S. Food and Drug Administration recognized AEW as a high-level water disinfectant [1,8]. China introduced AEW preparation technology in 1995, and 6 years later, the competent health authority issued a national standard for AEW production equipment, which was updated in 2020 to specify the definition, classification, physical properties, application scenarios, and usage methods of AEW [2,25]. Owing to its advantages of excellent disinfection efficacy, high safety, and environmental friendliness, AEW is widely applied in agricultural planting, livestock and poultry breeding, food processing, medical and health care fields [1,5,7,9,10,26,27]. In the healthcare sector, the main applications of AEW include the disinfection of flexible endoscopes, hygienic hands, skin, mucous membranes, water lines of dental units, and pre-sterilization medical devices [2]. The central sterile supply department (CSSD) is responsible for the cleaning, disinfection, and sterilization of reusable medical devices; in the disinfection process, AEOW is one of the primary methods alongside moist heat disinfection [6,28–31].

**Fig 1:**
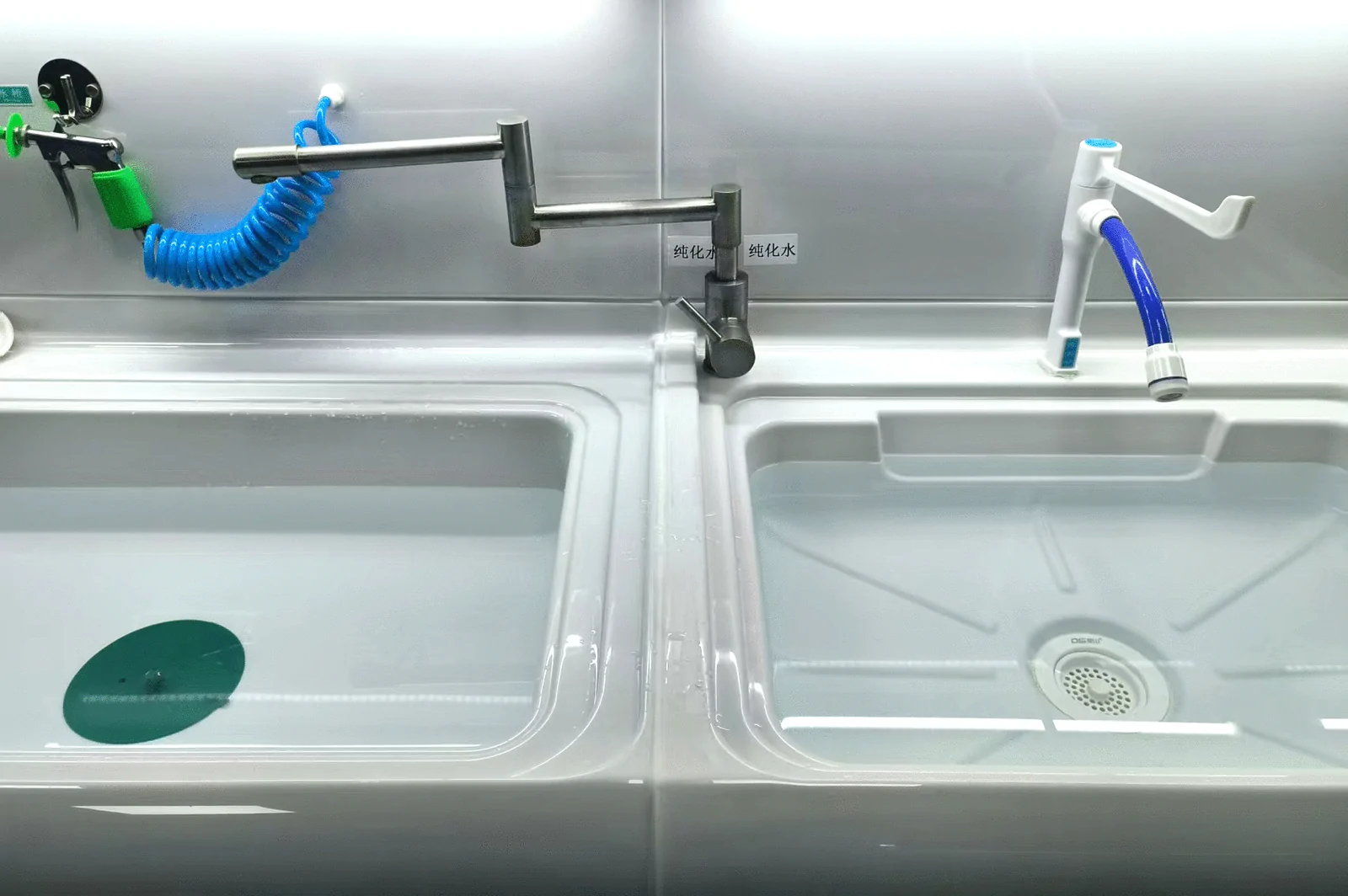
Visual comparison of acidic electrolyzed oxidizing water (AEOW) and purified water. Both AEOW (right) and purified water (left) appear as colorless, transparent liquids. The blue faucet on the right dispenses AEOW, while the central mobile stainless steel faucet is used to dispense purified water into both sinks.

The most common manual cleaning equipment in CSSDs is the manual cleaning station, which consists of multiple serially connected water tanks with distinct functions, undertaking separate processes such as pre-washing, washing, disinfection, and rinsing. The liquids required include tap water (for pre-washing and washing solution preparation), AEOW (for disinfection), and purified water (for rinsing) [30]. Due to space constraints in some CSSDs, taps for AEOW and either purified water or tap water are installed in the same tank, which may lead to misoperation—turning on purified water or tap water taps when AEOW is required for disinfection—thus failing to achieve the expected disinfection effect. Another common misoperation scenario involves CSSD technicians using movable small containers (e.g., plastic storage boxes, buckets) to hold AEOW for immersion disinfection of specific areas or medical devices. These containers need to be filled with AEOW at AEOW taps, but during operation, technicians may mistakenly dispense purified water or tap water, resulting in non-AEOW liquid in the containers and subsequent failure of medical device disinfection. Since AEOW, tap water, and purified water are all colorless and transparent liquids indistinguishable by the naked eye, and AEOW has only a faint chlorine odor that cannot be detected by technicians wearing face masks, these 2 types of misoperations are difficult to identify promptly after occurrence (Fig 1) [2,3]. Most importantly, AEOW solution is not replaced after single use but is used continuously in tanks for several hours; errors in the initial preparation of AEOW would invalidate all disinfection operations during this period, significantly amplifying the risk of infection. To mitigate these risks, operational standard operating procedures (SOPs) explicitly require testing the pH value and ACC of water at AEOW taps before each use to confirm that the liquid is qualified AEOW [2,25]. However, the practical implementation of this specification faces two major obstacles: first, the most commonly used methods for detecting pH and ACC are chemical test strip methods, which are highly subjective in result interpretation, cumbersome in operation, and generate additional medical waste; replacing these with electronic measuring instruments can improve detection accuracy but increases the economic and time costs associated with equipment purchase and regular calibration [32,33]. Second, prepared AEOW is typically used continuously for several hours (typically 2–4 hours), during which technician turnover and work task adjustments may occur; testing only before the first use cannot ensure that AEOW is not mistakenly replaced during subsequent use.

AEOW has high electrical conductivity (EC) because it is generated by the electrolysis of sodium chloride solution and contains large quantities of Na ^+^ , Cl ⁻ , H ^+^ , and ClO⁻ ions [5,34]. Considerable variations exist in the EC of AEOW generated by different brands and models of generators. Currently, no guidelines or standards specify the EC range of AEOW. Nevertheless, literature reports have documented that the EC of AEOW exceeds 1000 μS/cm [35–38]. This study conducted field tests on the EC of AEOW in 4 CSSDs, and the results were essentially consistent. The EC of AEOW is significantly higher than that of purified water (≤15 μS/cm) [30] and tap water from the vast majority of cities (approximately 200‑400 μS/cm) [39]. Against this background, this study developed the lighthouse‑shaped rapid identification device (LRID) for AEOW with an EC probe as the core component. The device enables technicians to promptly and accurately verify whether the disinfectant to be deployed is AEOW, thus preventing the misuse of tap water or purified water as AEOW within CSSDs. Meanwhile, the device features low cost and user‑friendly operation and does not substantially increase the financial and labor‑resource burdens of CSSDs.

## Materials and methods

### Device design and fabrication

**Fig 2:**
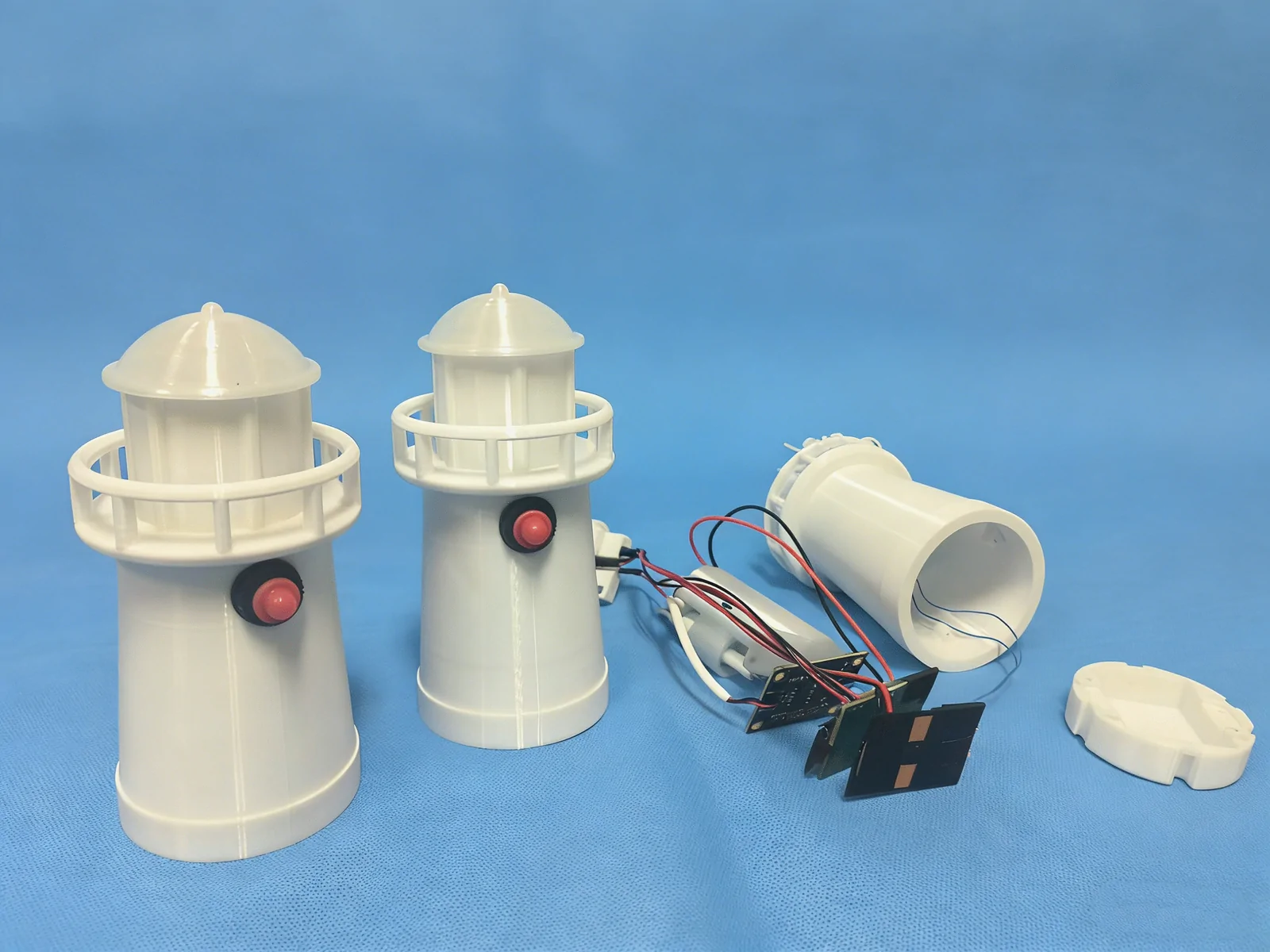
Lighthouse-shaped rapid identification device for AEOW.

**Fig 3:**
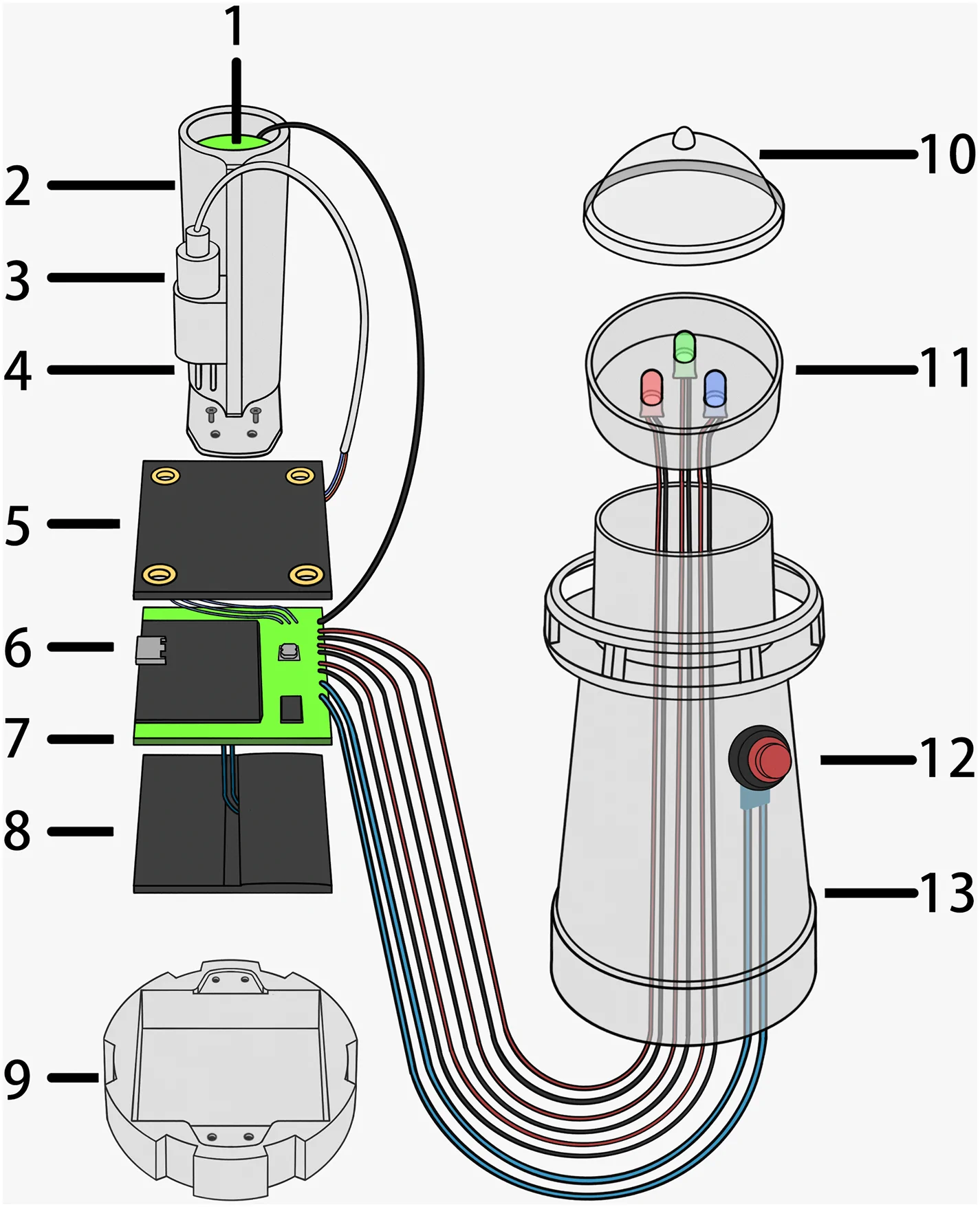
Schematic diagram of the lighthouse-shaped rapid identification device for AEOW. The battery (1) is housed within the battery compartment (2). The conductivity probe (3) is fixed to the side wall of the battery compartment, with the electrical conductivity sensor pin (4) suspended in space. The integrated microcontroller module (6) is mounted on the integrated control board based on a printed circuit board (7). The electrical conductivity signal adapter board (5), the integrated control board, and the wireless charging coil (8) are seated in the groove of the base (9). The translucent top cover (10) is positioned above the three LED indicator lights (11), and the power switch (12) is fixed at the center of the side wall of the lighthouse casing (13). Multiple irregular cut‑outs at the bottom are distributed around the base (9), serving as inflow‑outflow channels for the test solution.

The AEOW LRID was developed based on Arduino technology, and its hardware system consists of an integrated microcontroller module, an EC monitoring module, a battery and wireless charging module, a waterproof pushbutton, LED indicators, and a lighthouse-shaped enclosure (Figs 2 and 3). The device adopted an integrated printed circuit board (PCB) design with optimized spatial layout to enhance structural compactness and reliability of electrical connections. The PCB and wireless charging module are externally wrapped with nylon cloth-based tape, and all electrical components of the device are encapsulated with organic potting compound to achieve favorable protection and structural stability. Detailed parameters of the main components are as follows:

*Integrated microcontroller module:* The core is an Arduino Nano Mini-compatible development board (26 × 22 mm) with an Atmega 328P microcontroller as the main control unit. It integrates a Type-C interface and a CH340 USB-to-serial chip, supports 5 V DC input, and has a GPIO output voltage range of 3.3–5 V.

*EC monitoring module*: Composed of a signal adapter board (42 × 32 mm) and a dual-probe sensor, it supports 3.3–5.5 V DC input with an operating current of 3–6 mA. The probes have an effective length of 5 mm. The signal adapter board converts the water conductivity signal collected by the probe into an analog voltage signal ranging from 0 to 2.3 V.

*Battery and wireless charging module*: Power is supplied by a 3000 mAh, 18650 lithium-ion battery with a nominal output voltage of 3.7 V. The wireless charging module operates at a frequency of 100–200 kHz, outputting 5 V DC (max current 1 A, max power 5 W) after rectification and voltage regulation.

*Lighthouse-shaped enclosure*: Fabricated using 3D printing with PLA material, a wall thickness of 3 mm, a bottom diameter of 70 mm, and a height of 155 mm. The main body is white and opaque, while the top is translucent. Several irregular openings are preset at the bottom for the inflow and outflow of the test solution, and a waterproof pushbutton is installed on the side.

*Integrated PCB control board*: A customized PCB (42 × 33 mm) secures the main control module and hosts a boost converter, an LED driving circuit, and a battery charge-discharge management circuit.

The EC monitoring module is fixed inside the LRID, and the EC probe is mounted at a relatively low position, approximately 2 cm above the very bottom of the device. This arrangement ensures that the probe is constantly submerged beneath the AEOW liquid level. The liquid level of AEOW is required to be ≥ 5 cm, which is determined by the minimum height of stainless‑steel mesh baskets for holding medical devices; the AEOW level must exceed 5 cm to achieve full immersion of these baskets. One red, one blue, and one green LED are respectively installed under the translucent top enclosure of the LRID. The blue LED remains lit during charging and turns off automatically when fully charged. Upon pressing the waterproof button to power on, the red LED lights up for 2 s and then flashes 1–4 times according to the remaining battery level (representing approximately 25%, 50%, 75%, and 100% remaining power, respectively).

**Fig 4:**
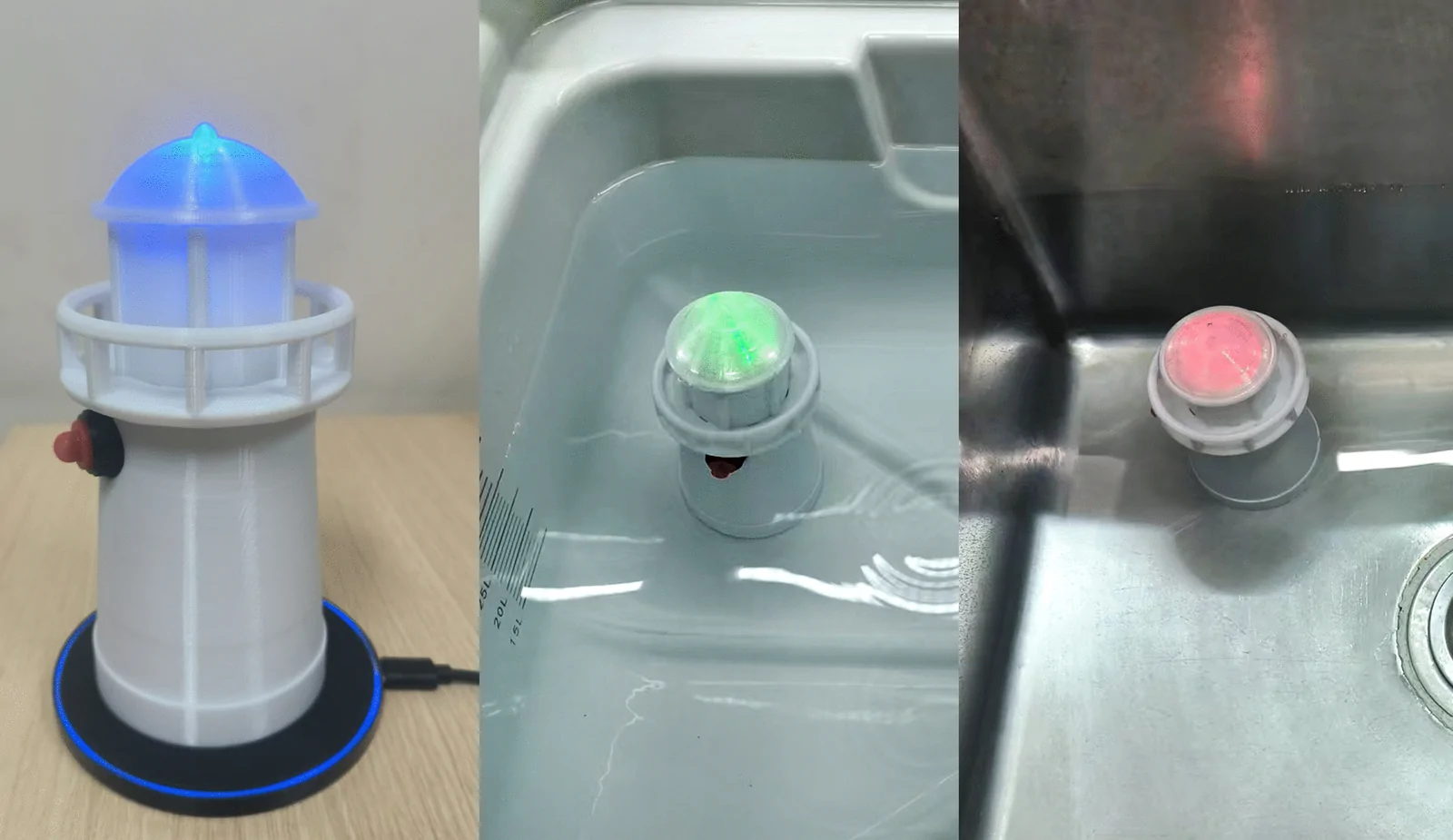
Three different working states of the lighthouse-shaped rapid identification device. It glows blue during charging, green in acidic electrolyzed oxidizing water, and red in tap water and purified water.

When the LRID is placed into the test solution, the liquid enters through the multiple irregular bottom‑located cut‑outs and fills the interior of the device. The signal collected by the conductivity probe is converted into an analog voltage signal by the adapter board, and the microcontroller converts the received analog voltage signal into an analog digital converter (ADC) value for threshold judgment accordingly. The LRID triggers different LED indicators according to distinct ADC values. In this study, the ADC discrimination threshold was set to 150: when the ADC value of AEOW exceeds 150, the green LED illuminates continuously, indicating that the liquid is AEOW and suitable for medical‑instrument disinfection. For tap water sourced from surface‑water supplies in most regions worldwide, its ADC value does not exceed 80, while purified water yields even lower ADC readings. Both liquids produce ADC values below the threshold of 150, which keeps the red LED steadily lit to signal that the solution is not AEOW and prohibits disinfection operations(Fig 4).

The threshold of 150 was selected after comprehensive trade‑offs among multiple factors. A total of three LRID prototypes were fabricated in this study. Considering that the minimum EC value of qualified AEOW is greater than 1000 μS/cm, two AEOW samples were diluted with purified water to adjust their EC to 1000 μS/cm, which were then tested using the three prototypes. The measured ADC values fluctuated within the range of 207‑212. Similarly, tap‑water samples collected from two hospitals in southern China (EC of 278 μS/cm and 288 μS/cm, respectively) were measured by the three LRID units, yielding ADC values ranging from 55 to 59. The EC of purified water is below 15 μS/cm, with corresponding ADC readings far lower than those of tap water. Theoretically, the discrimination threshold for LRID could be arbitrarily chosen within the range of 70‑200. The original experiments of this study were conducted in spring, which does not represent the coldest period throughout the year. Since the EC‑monitoring module of LRID has no temperature‑compensation function, EC will decrease synchronously as the solution temperature drops (approximately 2% reduction in EC per 1 °C temperature decrease). As CSSDs are indoor environments with strict temperature‑control requirements, the annual indoor temperature generally varies from 16 °C to 24 °C. Even under a 10 °C temperature drop, calculation based on the baseline ADC value of 207 shows that the ADC of AEOW would not drop below 160. Setting 150 as the discrimination threshold ensures sufficient safety margin. Within the acceptable range of safety margin, thresholds between 70 and 150 are all feasible. Nevertheless, a relatively higher value of 150 was adopted to mitigate false‑positive events caused by certain high‑conductivity cleaning agents. It is noteworthy that the tap‑water EC varies across cities, AEOW EC differs among hospitals, and the ADC span varies for different EC‑monitoring modules. Therefore, the optimal solution is for individual CSSDs to customize the threshold according to their on‑site conditions. Custom‑threshold configuration is straightforward and only requires modifying the threshold parameter in the software code.

In addition, the embedded program contains a low-battery protection function; when the residual power drops below 25%, the red LED keeps double-flashing to prompt users to recharge the device before further use. In practical, technicians turn on the LRID and place it into the sink or other container intended for AEOW. Once the green LED illuminates continuously within 2 s, subsequent disinfection procedures can proceed directly without additional monitoring of pH, ORP, or ACC. At the end of the workday, the LRID is removed and powered off for standby.

### Device validation

*Performance Validation:* Three performance verification tests were carried out on the LRID:

(1) Identification Accuracy: Three LRID units were selected to test AEOW, tap water, and purified water samples collected from the CSSDs of 10 hospitals. This verified the device’s ability to correctly identify AEOW (green LED) and exclude purified water or tap water (red LED).
(2) Battery Life: Three LRIDs were tested in both tap water and AEOW under continuous operation mode to assess battery endurance. Each device was tested 5 times in each type of liquid.
(3) Waterproof Performance: Three LRIDs were immersed in light-proof, sealed containers of AEOW for a soaking test. The AEOW was replaced every 3 days for a continuous immersion period of 30 days. Throughout the validation process, the ambient temperature was maintained at 18–24 ℃, with a relative humidity of 44–56%.
(4) Interfering‑liquid Identification: Five types of cleaning agents and one peracetic‑acid disinfectant solution commonly used in CSSDs were selected. Solutions with different dilution concentrations were prepared according to the manufacturer’s instructions. The EC values of each group of solutions were recorded, and three LRID units were used for discrimination tests to evaluate the identification accuracy for various interfering liquids. Detailed information on the six interfering liquids is presented in Table 1.

**Table 1:** Specifications, models, and recommended dilution concentrations of the six interfering liquids.

No.	Name	Manufacturer	Specification	Recommended dilution concentration
1	Prolystica 2X Concentrate Enzymatic Presoak and Cleaner	STERIS Corporation, USA	5 L per barrel	1:250-1:1000
2	Alkaline Detergent	Shinva Medical Instrument Co., Ltd., China	5 L per barrel	1:100-1:400
3	Endoscopic Multi-enzyme Low-foam Cleaner	Hangzhou Lvmon Medical Supplies Co., Ltd., China	1 L per bottle	1:200-1:800
4	High-Efficiency Alkaline Cleaner	Minnesota Mining and Manufacturing, USA	5 L per barrel	1:100-1:400
5	Universal High-Efficacy Multi-Enzyme Cleaning Solution	Minnesota Mining and Manufacturing, USA	5 L per barrel	1:100-1:400
6	Peracetic Acid Disinfectant	Shandong ANNJET Disinfection Technology Co., Ltd., China	Two‑component, Component A 250 mL, Component B 250 mL	1:30-1:300

*Technician Satisfaction*: A total of 28 CSSD technicians from 3 hospitals, who had used the LRID, were invited to rate their satisfaction with the pH test strip method versus the LRID for AEOW determination. The survey instrument was a self-designed satisfaction questionnaire consisting of three items: Identification accuracy, ease of operation, and promotability. Each item was scored using a 5-point Likert scale (1 = very dissatisfied, 2 = dissatisfied, 3 = moderately satisfied, 4 = satisfied, 5 = very satisfied).

### Statistical analysis

R software (version 4.5.1) was used for data analysis. Count data were described using frequencies and percentages (%). Measurement data that did not conform to a normal distribution were expressed as M (P_25_, P_75_). Comparisons between the two methods were performed using the Wilcoxon signed-rank test. A value of P < 0.05 was considered statistically significant.

## Results

### Identification accuracy verification

All 3 LRID units accurately identified AEOW, tap water, and purified water samples collected from the CSSDs of 10 hospitals, achieving an identification accuracy of 100% (90/90).

### Battery life verification

The 3 LRIDs completed 15 battery endurance tests in both AEOW and tap water. The mean battery life was 83.75 ± 1.27 h.

### Waterproof performance verification

After continuous immersion in AEOW for 30 days, all 3 LRID units remained fully functional with no abnormalities.

### Interfering‑liquid identification verification

At the dilution concentrations recommended by the manufacturer’s instructions, two of the five cleaning agents and the peracetic‑acid disinfectant among the six interfering liquids exhibited high EC. Their ADC values exceeded the LRID discrimination threshold, leading to false‑positive outputs (misclassified as qualified AEOW). For the remaining three cleaning agents at their recommended dilution concentrations, the LRID activated the red LED, achieving correct identification (Table 2).

**Table 2:** Discrimination results of the LRID for six interfering liquids at different dilution concentrations.

Solution	Recommended dilution concentration	Test 1			Test 2			Test 3			Test 4		
		Concentration	EC (μS/cm)	LED indication	Concentration	EC (μS/cm)	LED indication	Concentration	EC (μS/cm)	LED indication	Concentration	EC (μS/cm)	LED indication
A	1:250-1:1000	1:200	330	Red	1:300	202	Red	1:400	171	Red	1:500	135	Red
B	1:100-1:400	1:100	1040	Green	1:200	510	Red	1:300	322	Red	1:400	211	Red
C	1:200-1:800	1:200	919	Green	1:300	601	Red	1:400	460	Red	1:500	384	Red
D	1:100-1:400	1:100	529	Red	1:200	251	Red	1:300	176	Red	1:400	142	Red
E	1:100-1:400	1:100	520	Red	1:200	245	Red	1:300	169	Red	1:400	125	Red
F	1:30-1:300	1:30	2063	Green	1:100	870	Green	1:200	538	Red	1:300	394	Red

A: Prolystica 2X Concentrate Enzymatic Presoak and Cleaner.

B: Alkaline Detergent.

C:Endoscopic Multi-enzyme Low-foam Cleaner.

D: High-Efficiency Alkaline Cleaner.

E: Universal High-Efficacy Multi-Enzyme Cleaning Solution.

F: Peracetic Acid Disinfectant.

### Technician satisfaction

The total satisfaction score of 28 CSSD technicians for the LRID, as well as the scores across the 3 dimensions of identification accuracy, ease of operation, and promotability were all higher than those for the pH test strip method (Table 3), with statistically significant differences (P < 0.001).

**Table 3:** Comparison of CSSD technicians’ satisfaction with two AEOW identification methods (n = 28, M, (P_25_,P_75_)).

Methods	Total satisfaction score	Identification accuracy	Ease of operation	Promotability
pH test paper	10(9,11)	4(3,4)	3(2,3)	3.5(3,4)
LRID	14(13,15)	5(5,5)	4(3.75,5)	5(5,5)
V value	0	0	0	0
P value	<0.001	<0.001	<0.001	<0.001

## Discussion

The lighthouse-shaped design of the LRID was primarily driven by considerations of structural practicality and symbolic significance. First, the top of a lighthouse is naturally designed to carry indicator lights, a structure that aligns highly with the application scenario of the LRID. The LRID is placed in the sink at approximately waist height for technicians, and mounting the LED indicators at the top facilitates clear observation. The surface of the lighthouse enclosure is smooth with no obvious grooves, making it easy to clean and preventing the accumulation of dirt. Second, the lighthouse features a narrow-top and wide-bottom structure. Placing internal components at the bottom increases the weight of the base, allowing the device to stand stably at the bottom of the sink. Even when the sink is full and water turbulence occurs, the LRID will not tip over. The LRID can be steadily placed in the corner of the sink, reducing space occupation and minimizing disruption to technicians’ work. The interior of the lighthouse provides a large effective volume, supporting reasonable layout of internal components with regular space and high space utilization. Finally, the lighthouse carries a clear symbolic meaning of guidance and judgment, which aligns well with the LRID’s purpose of rapid identification and accurate determination.

The LRID performs discrimination solely by detecting solution EC, a strategy identified as optimal following a comprehensive evaluation of multiple alternative indicators. The key indicators of AEOW are pH 2–3, ORP ≥ 1100 mV, and ACC 50–70 mg/L[2, 6]. While all 3 differ significantly from tap water and purified water and could theoretically serve as the basis for LRID determination, they were not adopted due to specific drawbacks. pH probes are relatively bulky; common pH probes exceed 15 cm in length, and the signal adapter board is 2 cm in height, making them impossible to install inside the current lighthouse-shaped enclosure. Accommodating a pH probe would require increasing the LRID height by 10 cm and enlarging the base area, resulting in a significantly increased overall volume. This would occupy excessive sink space and hinder workflow. In addition, a pH probe costs approximately 8 USD, considerably higher than the 2 USD of an EC probe, and requires regular maintenance. However, the fabricated LRID is fully potted and does not support probe disassembly for maintenance. ORP probes are even larger (17 cm) and more expensive (30 USD), and also require periodic maintenance. Therefore, neither pH nor ORP probes are suitable for the LRID. Commercial sensors for direct ACC measurement are currently unavailable; instead, quantification relies on iodometric titration [40–42] or test strip methods [6], neither of which can be easily converted into electrical signals for LRID determination. In contrast, EC probes are inexpensive, compact, and require virtually no maintenance (no membranes or electrolytes). Meanwhile, EC reflects the total ion concentration in the liquid. Although pH, ORP, and ACC of AEOW may change considerably during storage and use, EC remains highly stable with minimal variation [43]. Therefore, using EC for rapid AEOW identification represents the optimal strategy. Since this study aims to facilitate rapid on-site identification of AEOW and improve workflow efficiency rather than precisely measure parameters like pH or ORP, employing EC as the sole indicator is both reasonable and feasible.

The stability and reliability of the LRID basically meet the long-term application requirements of CSSDs. Identification accuracy forms the foundation for the LRID’s core function of rapid AEOW identification. All 3 LRIDs correctly identified AEOW, tap water, and purified water from 10 CSSDs with 100% accuracy, which is attributed to the distinct EC differences among the 3 solutions. Battery life and waterproof performance are critical for supporting long-term device operation. The LRID demonstrated a battery life of 83.75 ± 1.27 h with a narrow fluctuation range, indicating a stable power supply system that reduces charging frequency and alleviates the operational burden on technicians. After continuous immersion in AEOW for 30 days, all LRIDs remained fully functional with no abnormalities, fully verifying reliable sealing performance. Combined with the favorable water resistance and chemical corrosion resistance of PLA material, the LRID can adapt to the long-term humid, AEOW-exposed environment of CSSD sinks, avoiding device failure caused by water intrusion or corrosion and ensuring long-term stable operation.

In the interfering‑liquid identification validation test, the LRID produced false‑positive outcomes for two cleaning agents and one peracetic‑acid diluent prepared at the working concentrations recommended by the manufacturer’s instructions. This phenomenon occurs because certain cleaning agents and peracetic acid contain high levels of electrolytes, which confer high EC to their diluted solutions and may compromise the practical performance of the LRID. Nevertheless, this limitation should not be over‑emphasized. The solutions that trigger false‑positive readings have distinct sensory properties compared with AEOW. The cleaning agents appear light yellow, light green, or light blue, which allows visual differentiation from AEOW. Peracetic acid exhibits a strong acidic odor that is perceptible even with a face mask worn, enabling easy distinction from AEOW. Therefore, even though some cleaning agents and peracetic acid may cause misjudgment by the LRID, differences in sensory characteristics, together with clinical workflow arrangements where AEOW and cleaning‑agent solutions are never used in the same sink, prevent operators from placing the LRID into diluted cleaning‑agent solutions. In the rare scenario where the LRID is accidentally immersed in diluted peracetic‑acid solution and misidentified as AEOW, the associated clinical risk is negligible. Both peracetic acid and AEOW are high‑level disinfectants capable of achieving medical‑device disinfection within several minutes.

CSSD technicians expressed higher satisfaction with the LRID than with the pH test strip method. The pH test strip method was selected as the control for evaluating technician satisfaction. As a low-cost, simple, and widely used on-site identification method in CSSDs that can be correctly performed by all technicians, it serves as a representative and targeted control [6]. Despite its widespread use, the pH test strip method has obvious limitations: it requires manual sampling and visual reading, with results strongly affected by subjective judgment and ambient lighting, and the entire process takes more than 1 minute. In addition, used pH test strips must be discarded promptly to avoid staining work surfaces, increasing additional cleaning workload. In comparison, the LRID offers significant advantages. It can be placed directly in the sink corner,enabling rapid, real-time AEOW identification via a continuously illuminated top LED. This process requires no manual intervention and imposes no extra workload. Identification results are instant, intuitive, and clear, with negligible additional time cost. The device is straightforward to operate and can be deployed without specialized training, resulting in an extremely low learning curve. A single weekly charge suffices for operation, and the conductivity probe requires no calibration or maintenance, further reducing usage burdens. Economically, the LRID is low-cost, with a total unit cost of approximately 20 USD (3D-printed enclosure: 10 USD; battery and wireless charging module: 4 USD; integrated microcontroller module and PCB: 3 USD; EC monitoring module: 2 USD; LEDs, switches, and other components: 1 USD). The LRID outperforms the pH test strip method in identification accuracy, ease of operation, and promotability (P < 0.001), thereby achieving higher satisfaction ratings from CSSD technicians.

### Limitations and future work

The LRID was developed based on the premise that the EC of AEOW is markedly higher than that of tap water and purified water. Nevertheless, in some regions (e.g., South Asia, the western United States, and North China), municipal tap water is sourced from groundwater with high EC values that can approach or even exceed 1000 μS/cm, rendering the LRID inapplicable. To mitigate this limitation, future work plans to validate discrimination using a pH sensor, while comprehensively considering the impacts of increased cost, changes in device size or form factor, and sensor‑calibration requirements. Furthermore, adoption of a pH sensor would completely eliminate false‑positive readings induced by high‑conductivity cleaning agents.

The current monolithic lighthouse structure precludes rapid sensor removal. Considering that the EC probe may require replacement during long-term service, future iterations will adopt a detachable modular design with an externalized probe. This configuration will not only facilitate probe replacement but also allow the main unit to be positioned on the sink edge, avoiding continuous immersion in AEOW. Consequently, this will lower waterproofing requirements and extend the service life. Furthermore, should a pH sensor be adopted in the future, this modular structure would avoid increasing in the device’s volume and facilitate sensor maintenance and calibration.

## Conclusion

This study developed an LRID for AEOW based on the EC differences among AEOW, tap water, and purified water. Systematic experiments verified its high identification accuracy, demonstrating robust battery life and waterproof performance that meet the demands of long-term on-site use in CSSDs. With significant advantages including ease of operation, real-time feedback, time efficiency, no requirement for specialized training, and low maintenance costs, the LRID represents a low-cost and rapid AEOW identification method. It has received high recognition from CSSD technicians and holds promising application prospects.

## Supporting information

S1 Data Research data. (XLSX)

S2 Data Human_Participants_Research_Checklist. (PDF)
